# Rapid Antigen Diagnostics as Frontline Testing in the COVID‐19 Pandemic

**DOI:** 10.1002/smsc.202200009

**Published:** 2022-07-05

**Authors:** Jiang Xu, Liam Kerr, Yue Jiang, Wenhao Suo, Lei Zhang, Taotao Lao, Yuxin Chen, Yan Zhang

**Affiliations:** ^1^ Department of Systems Biology Blavatnik Institute Harvard Medical School Boston MA 02115 USA; ^2^ Department of Molecular Virology Virogin Biotech Ltd. 3800 Wesbrook Mall Vancouver BC V6S 2L9 Canada; ^3^ Department of Mechanical Engineering Center for Intelligent Machines McGill University Montreal QC H3A0C3 Canada; ^4^ China-Australia Institute for Advanced Materials and Manufacturing Jiaxing University Jiaxing 314001 China; ^5^ Dana-Farber Cancer Institute Harvard Medical School Boston MA 02215 USA; ^6^ Department of Pathology The First Affiliated Hospital of Xiamen University 55 Zhenhai Road Xiamen 361003 China; ^7^ Department of Chemical Engineering Waterloo Institute for Nanotechnology University of Waterloo 200 University Avenue West Waterloo ON N2L3G1 Canada; ^8^ Department of Molecular Diagnostics Boston Molecules Inc. 564 Main Street Waltham MA 02452 USA; ^9^ Center for Immunology and Inflammatory Diseases Massachusetts General Hospital Harvard Medical School Charlestown MA 02114 USA; ^10^ Department of Laboratory Medicine Nanjing Drum Tower Hospital Nanjing University Medical School Nanjing Jiangsu 210008 China; ^11^ Tianjin Key Laboratory for Modern Drug Delivery and High-Efficiency Collaborative Innovation Center of Chemical Science and Engineering School of Pharmaceutical Science and Technology Tianjin University Tianjin 300072 China; ^12^ Frontiers Science Center for Synthetic Biology (Ministry of Education) Tianjin University Tianjin 300072 China

**Keywords:** antigen tests, COVID-19, in vitro diagnostics, immnuoassays

## Abstract

The ongoing global COVID‐19 pandemic, caused by the SARS‐CoV‐2 virus, has resulted in significant loss of life since December 2019. Timely and precise virus detection has been proven as an effective solution to reduce the spread of the virus and to track the epidemic. Rapid antigen diagnostics has played a significant role in the frontline of COVID‐19 testing because of its convenience, low cost, and high accuracy. Herein, different types of recently innovated in‐lab and commercial antigen diagnostic technologies with emphasis on the strengths and limitations of these technologies including the limit of detection, sensitivity, specificity, affordability, and usability are systematically reviewed. The perspectives of assay development are looked into.

## Graphical abstract

Antigen test kits play important roles in the frontline of COVID‐19 testing because of their convenience, low cost, and high accuracy. Herein, different types of recently innovated in‐lab and commercial antigen diagnostic technologies with emphasis on the strengths and limitations of these technologies including the limit of detection, sensitivity, specificity, affordability, and usability are systematically reviewed.

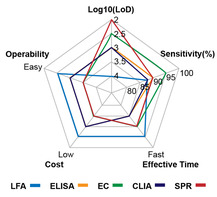

## 1 Introduction

**Figure 1:**
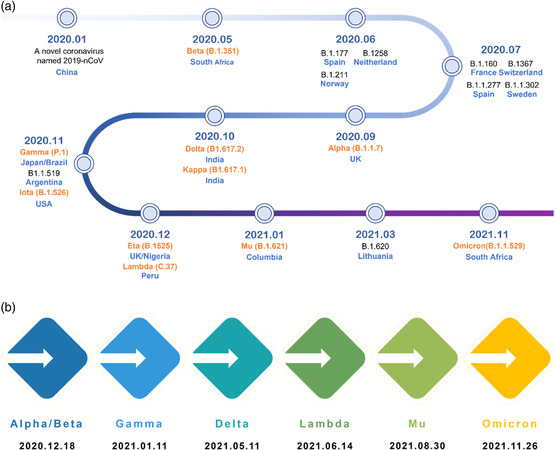
Timeline of the emergence of SARS‐CoV‐2 variants and those of greater concern.^[^ ^116^ ^]^ a) Timeline of the emergence of SARS‐CoV‐2 variants. All variants are presented with corresponding first‐identified date, pango lineage, and first‐identified location, ten of which (labeled in orange) have been named by WHO using letters of the Greek Alphabet as of December 2021. b) Date of designation of variants of concern by WHO.

The SARS‐CoV‐2 virus‐caused COVID‐19 pandemic has resulted in an unprecedented public health disaster and devastating economic damage. SARS‐CoV‐2 infection can cause acute damage to various organs,^[^ 1, 2, 3 ^]^ particularly the lungs,^[^ 2, 3, 4 ^]^ and can be fatal in the elderly and those who with heart or lung conditions, weakened immune systems, obesity, or diabetes. Long‐lasting sequelae symptoms including dyspnea, depression, muscle weakness, mobility impairment, etc. in survivors have also been recorded in longitudinal cohort studies.^[^ 5, 6 ^]^ Since the outbreak of the COVID‐19 pandemic in December 2019, dozens of variants with distinct strain clades have arisen globally (**Figure** **1**a), seven of which were labeled and classified as variants of concern or interest by World Health Organization (WHO)^[^ ^7^ ^]^ as of April 12, 2022 (Figure 1b). Despite the recent development of COVID‐19 vaccines,^[^ 8, 9 ^]^ the lower‐than‐expected vaccination rates,^[^ 10, 11 ^]^ fast waning vaccine effectiveness,^[^ ^12^ ^]^ and ascending demand of booster doses^[^ 13, 14 ^]^ make the progression of the pandemic difficult to predict. Furthermore, despite their effectiveness in reducing the rates of severe diseases and mortality, none of the available vaccinations have been demonstrated to be effective in stopping the spread of COVID‐19. Therefore, timely detection of COVID‐19 is extremely important for monitoring the scale of infection and for ensuring early treatment.

**Figure 2:**
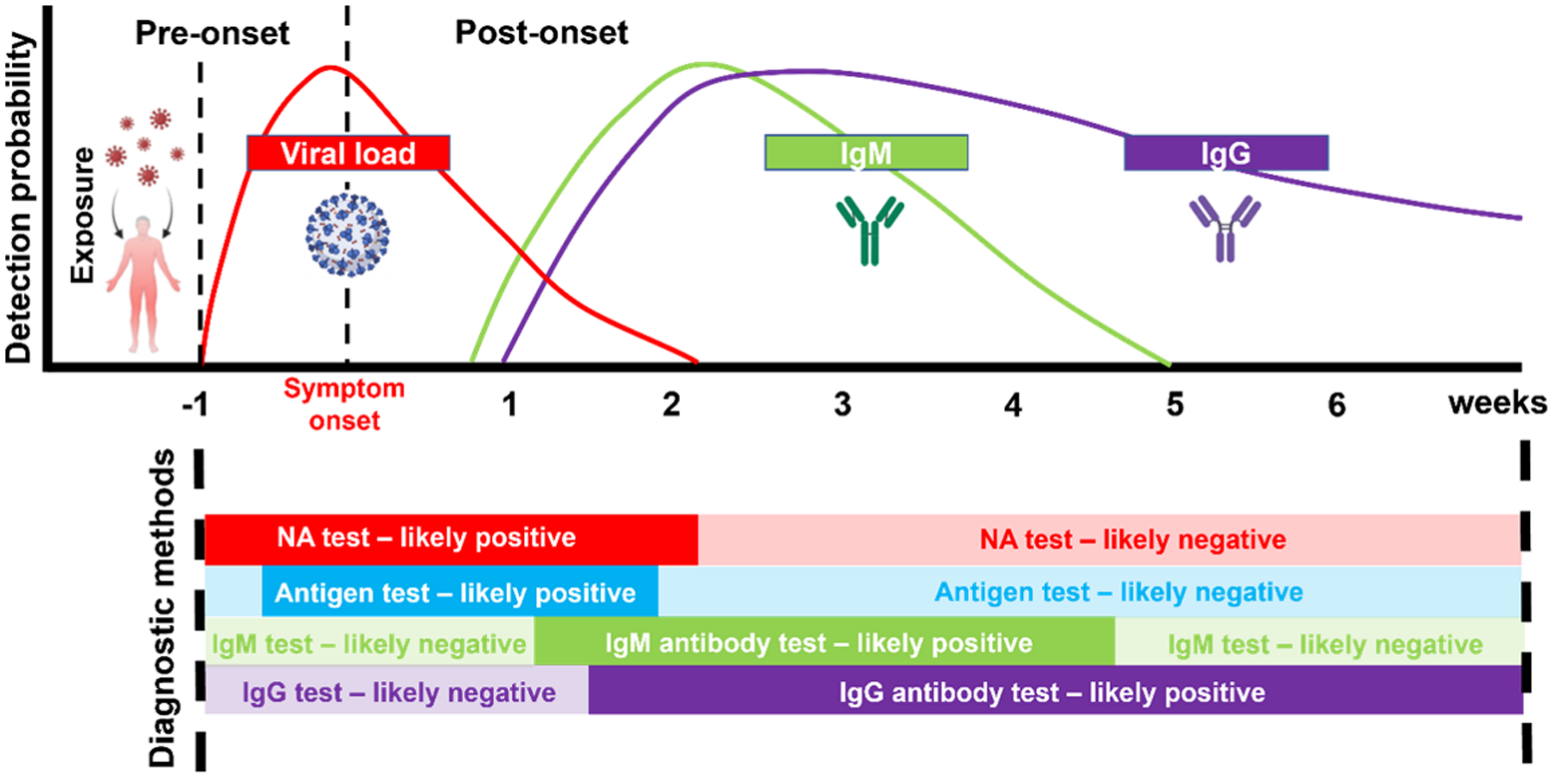
Estimated viral load and immune responses before and after symptom onset^[^ 106, 117, 118, 119 ^]^ and appropriate diagnostic methods at different phases of infection. This figure was created with BioRender.com.

This perspective focuses on the antigen test because it offers distinct advantages over other diagnostic approaches such as the nucleic acid (NA) test and the antibody test. NA tests in general employ polymerase chain reaction (PCR) technology, requiring repetitive temperature control steps for signal amplification, and thus are time‐consuming and instrument dependent.^[^ ^15^ ^]^ In contrast, antigen tests are quick and in expensive, with some of them simple to operate, making them suitable for the purposes of use in‐home, at the point of care (POC), and even self‐testing.^[^ ^16^ ^]^ So far antigen tests have been broadly used and proven to be able to greatly alleviate the testing demands when PCR resources are saturated due to prematurely relaxing lockdown measures in many countries.^[^ ^17^ ^]^ In addition, antigen tests are effective in detecting asymptomatic infections and provide valid results prior to symptom onset for symptomatic infections, which is in contrast to antibody tests that are 1–2 weeks delayed in response (**Figure** **2**).

The omicron variant has become the prevalent strain in most nations since the start of year 3 of the COVID‐19 pandemic. It was fortunate that the measured case fatality rate had fallen considerably, most likely due to the protection of vaccination and the efficacy of new antiviral drugs. As the public is weary and longs for a return to normal, many countries have lifted the lockdown ignoring the fact that omicron is a highly transmissible variant with an average relative *R*_0_ (basic reproduction number) to Delta as 2.5.^[^ ^18^ ^]^ As a result, the number of infected people soared, quickly overwhelming the NA testing capacity at many places. Under such backgrounds, commercial antigen test kits especially self‐testing kits were approved for emergency use around the world. More recently excess mortality rate was used to evaluate the damage to the community by Omicron,^[^ 19, 20 ^]^ highlighting the importance of tighter pandemic control and more frequent testing of potential viral exposure. Up to the date of this manuscript in preparation, more than 1000 commercial antigen test kits have been available in Europe and the United States. We have performed a thorough review of these commercial kits devoting attention to their targets and testing principles, statistically comparing their performance and application scenarios, hoping to guide the choice and use of antigen tests and more importantly to direct future development of pandemic control policy and testing technology.

## 2 Targets of SARS‐CoV‐2 Antigen Detection

**Figure 3:**
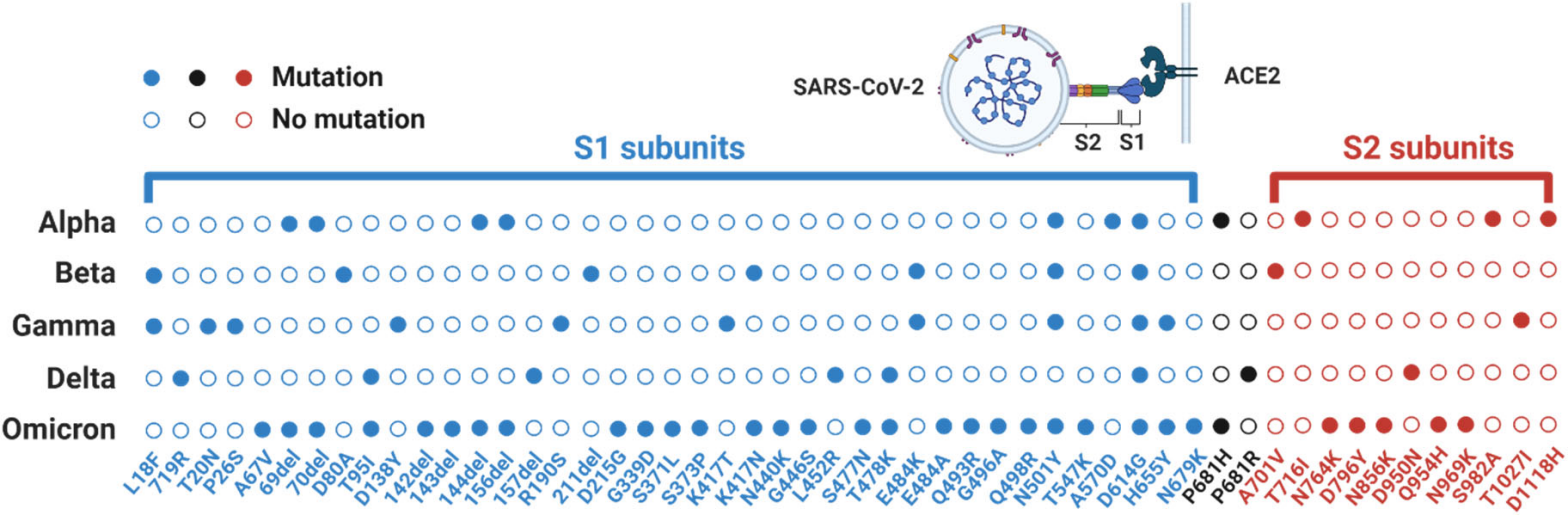
Mutations of S proteins in different SARS‐CoV‐2 variants. Mutations are called in reference to Wuhan‐Hu‐1 (NCBI Reference Sequence: NC 045512.2).^[^ 120, 121 ^]^ S1 and S2 subunits are labeled in blue and red, respectively.

The genome size of SARS‐CoV‐2 is 29.6 kilobase, which only shares 79.0% sequence identity to SARS‐CoV^[^ ^21^ ^]^ and thus contributes to the unique pathogenicity of SARS‐CoV‐2.^[^ ^22^ ^]^ The SARS‐CoV‐2 contains 16 nonstructural proteins (NSP), 9 other accessory factors, and 4 structural proteins.^[^ ^23^ ^]^ Four structural proteins, including spike (S), envelope (E), membrane (M), nucleocapsid (N), are composed of 1273, 75, 222, and 419 amino acids, respectively.^[^ ^24^ ^]^ The S protein of SARS‐CoV‐2 is a trimeric glycoprotein consisting of two subunits (S1 and S2) upon cleavage by the host protease, responsible for attachment and fusion of viral and cellular membranes through angiotensin‐converting enzyme 2 (ACE2) of the host cells, respectively^[^ ^25^ ^]^ (**Figure** **3**). The important physiological role and abundance of S protein potentiate a good target in antigen diagnostic tests. However, it was soon realized that the S protein contains mutation hotspots. These mutations even caused significant changes of the overall protein structure^[^ 26, 27 ^]^ that are in turn responsible for altered antigenic properties. Taken together, it is not practical to develop an anti‐S antibody‐based antigen test kit that could be applied to detect all variants from Alpha to Omicron and the next new variants (Figure 3). To date, most COVID‐19 test kits, especially commercial ones, target the N protein, which is the most conserved protein among all four structural proteins and is more abundant than the E and M proteins^[^ ^28^ ^]^ (**Table** **1**).

**Table 1:** Mutated amino acid count of Omicron variant (BA.1) by comparing with Wuhan‐Hu‐1 (NCBI Reference Sequence: NC 045512.2) reference sequence.^[^ 120, 121 ^]^

Structural proteins	Mutational counts [amino acid]	Mutation rates [normalized by length]
Spike (S)	32	2.51%
Envelope (E)	1	1.33%
Membrane (M)	3	1.35%
Nucleocapsid (N)	4	0.95%

## 3 Principles of SARS‐CoV‐2 Antigen Tests

**Figure 4:**
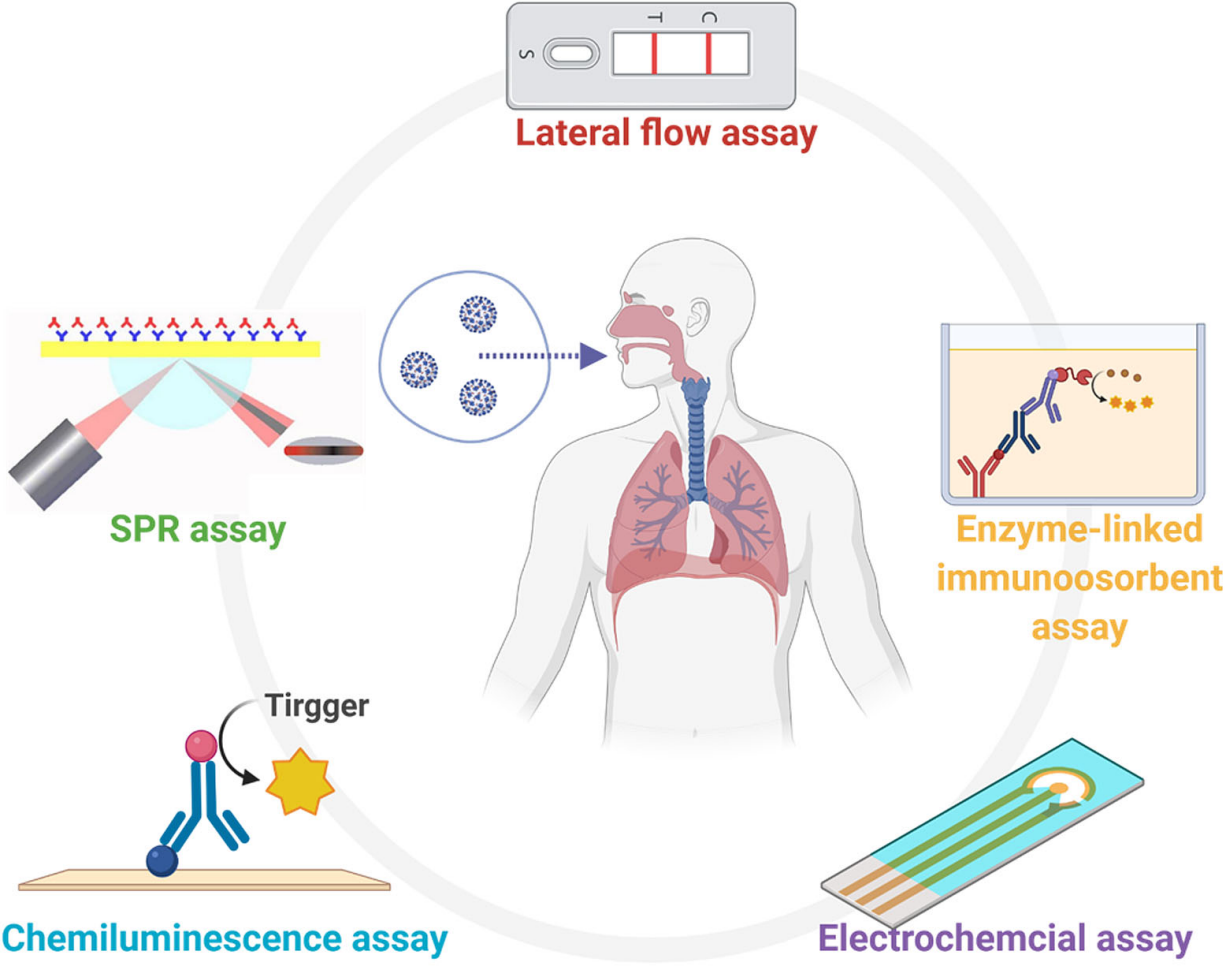
Common SARS‐CoV‐2 viral antigen diagnostic methods based on different mechanisms. This figure was created with BioRender.com.

Based on the testing principles, the existing Sars‐CoV‐2 antigen tests can be categorized into lateral flow assay (LFA), enzyme‐linked immunosorbent assay (ELISA), chemiluminescence assay (CLIA), electrochemical assay, and surface plasmon resonance (SPR) assay (**Figure** **4**).

## 4 Lateral Flow Assay (LFA)

**Figure 5:**
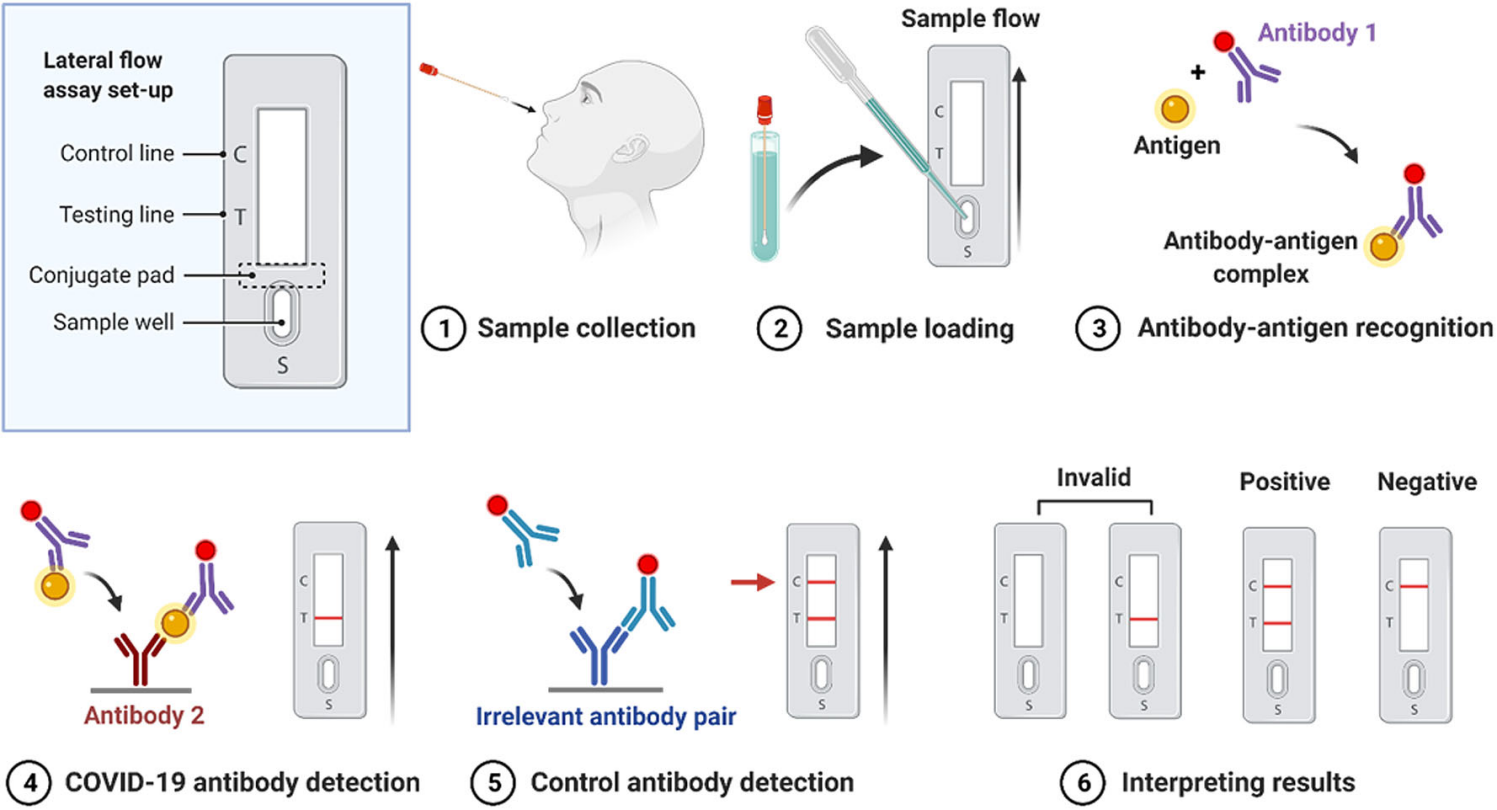
Schematic of testing workflow and mechanism of a typical LFA, created with BioRender.com.

LFA is a multilayered paper‐like substrate with functional components including a sample well, a conjugate pad, and a nitrocellulose membrane featured with testing and control lines (**Figure** **5**). In a typical LFA test, buffer solution contains lysing components (i.e., Triton X‐100) to decompose the viruses in a collected sample down to small antigen fragments (Figure 5 Step 1), which reduces steric hindrance of target antigen sites and thus facilitates subsequent antigen binding. Sample solution is added to the sample pad and flows toward the conjugated pad (Figure 5 Step 2), where gold nanoparticles conjugated to a specific COVID‐19 antibody (Ab 1) are embedded. The antigens in a positive sample bind to the Ab‐1‐conjugated nanoparticles and form complexes (Figure 5 Step 3), which continue to migrate and are immobilized by another antibody (Ab 2) at the test line (Figure 5 Step 4). An irrelevant antibody pair is often employed with one conjugated to gold nanoparticles and the other at the control line (Figure 4 Step 5 and 6).

The limit of detection (LoD) of LFAs are reported to be around 10^3^–10^4^ viral copies mL^−1^ (equivalent to a Ct value in the range of 20–30 in qPCR assay),^[^ 29, 30, 31 ^]^ which is considered to be a viral load with relatively low risk of transmission.^[^ ^32^ ^]^ Fluorescent dyes are also commonly used for higher sensitivity of LFAs.^[^ ^33^ ^]^ SARS‐CoV‐2 LFA is valuable as an alternative solution to NA testing for large‐scale screening due to its easy operation, low cost, and fast readout. Its accuracy has also been recognized by certain regions in the implementation of public health and travel policies.^[^ 34, 35, 36 ^]^ The major disadvantage of LFA lies in its relative low sensitivity compared with NA testing. In addition, color appearance and intensity is based on subjective perception and thus a significant difference in test sensitivity was found between professional and self‐trained users.^[^ ^37^ ^]^ Smartphones^[^ 38, 39, 40 ^]^ and artificial intelligence^[^ 41, 42 ^]^ have been employed to overcome this limitation by improving result interpretation and data collection.

## 5 Enzyme‐Linked Immunosorbent Assay (ELISA)

**Figure 6:**
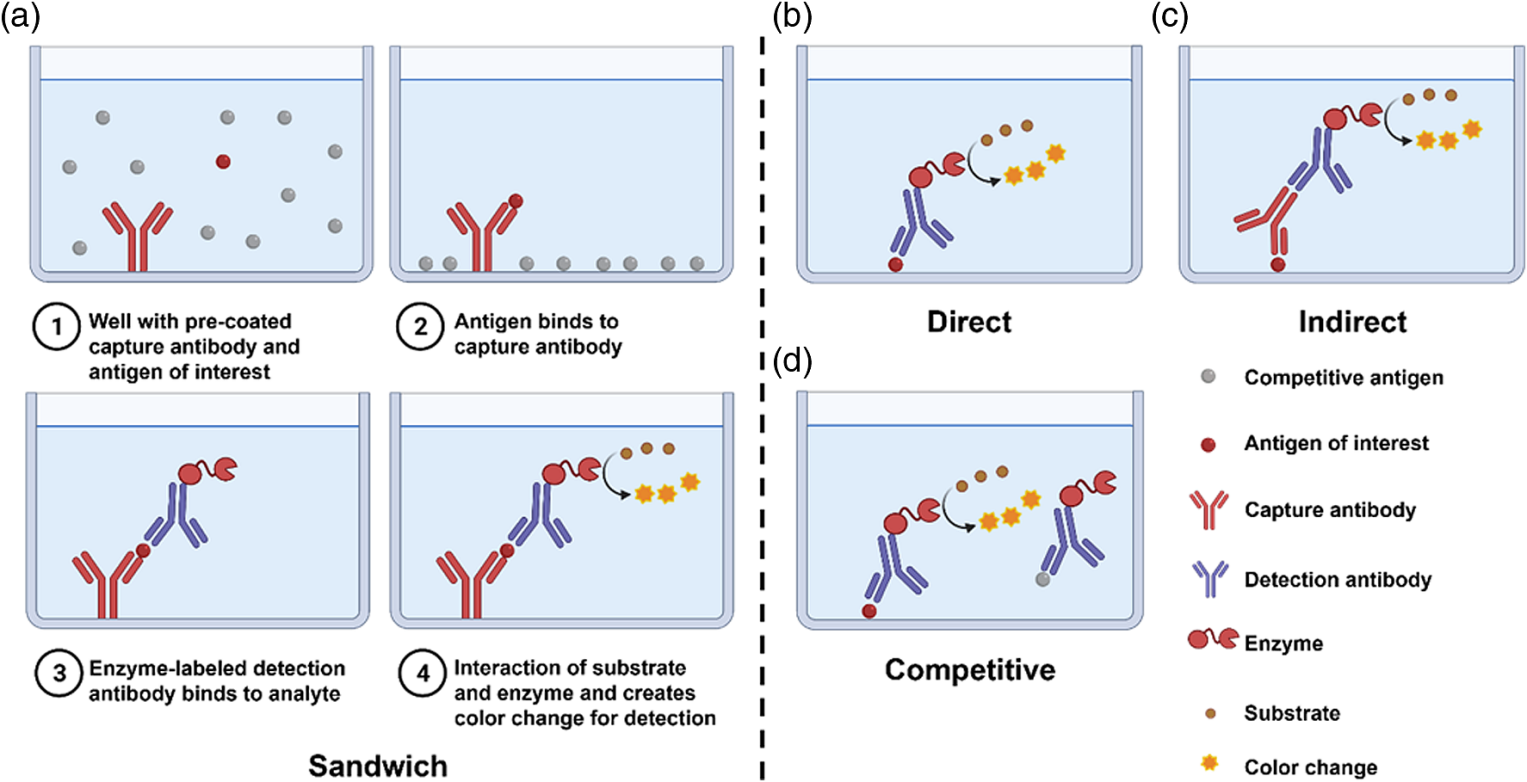
Basic setup and procedures of sandwich ELISA and principles of direct, indirect, and competitive ELISAs. This figure was created with BioRender.com.

ELISA is a common assay in analytical biochemistry, first described by Engvall and Perlmann in 1971,^[^ ^43^ ^]^ particularly for soluble protein targets like peptides,^[^ ^44^ ^]^ antibodies,^[^ ^45^ ^]^ and hormones.^[^ ^46^ ^]^ ELISA detects analyte through a specific antigen–antibody interaction and a subsequent color or fluorescence signal generated from enzyme–substrate reaction^[^ ^47^ ^]^ (**Figure** **6**). The sandwich ELISA method is particularly suitable for the detection of unconcentrated targets in solution and is therefore the most widely used ELISA method for detection of COVID‐19 antigens.^[^ ^48^ ^]^ ELISA test can either qualitatively or quantitatively identify viral copies on the scale of 10^3^ viral copies mL^−1^.^[^ ^48^ ^]^ Such sensitivity is close to the PCR‐based NA tests^[^ ^48^ ^]^ and better than typical LFAs.^[^ ^49^ ^]^ Moreover, the signal intensities(absorbance or fluorescence) of ELISA are well correlated with the concentrations of the analyte.^[^ 50, 51 ^]^ Thus, it has been used as a standard tool for antibody screening^[^ 52, 53 ^]^ and methodology evaluation.^[^ 54, 55 ^]^ Nevertheless, the excellent LoD and sensitivity of ELISA are equipment dependent requiring a fluorescent/color signal reader,^[^ ^56^ ^]^ which inevitably increases the complexity of operation and time to result (typically 1−5 h^[^ ^57^ ^]^), restricting its application in point of care testing (POCT) scenarios.^[^ ^55^ ^]^

## 6 ChemiLuminescent Immunoassay (CLIA)

**Figure 7:**
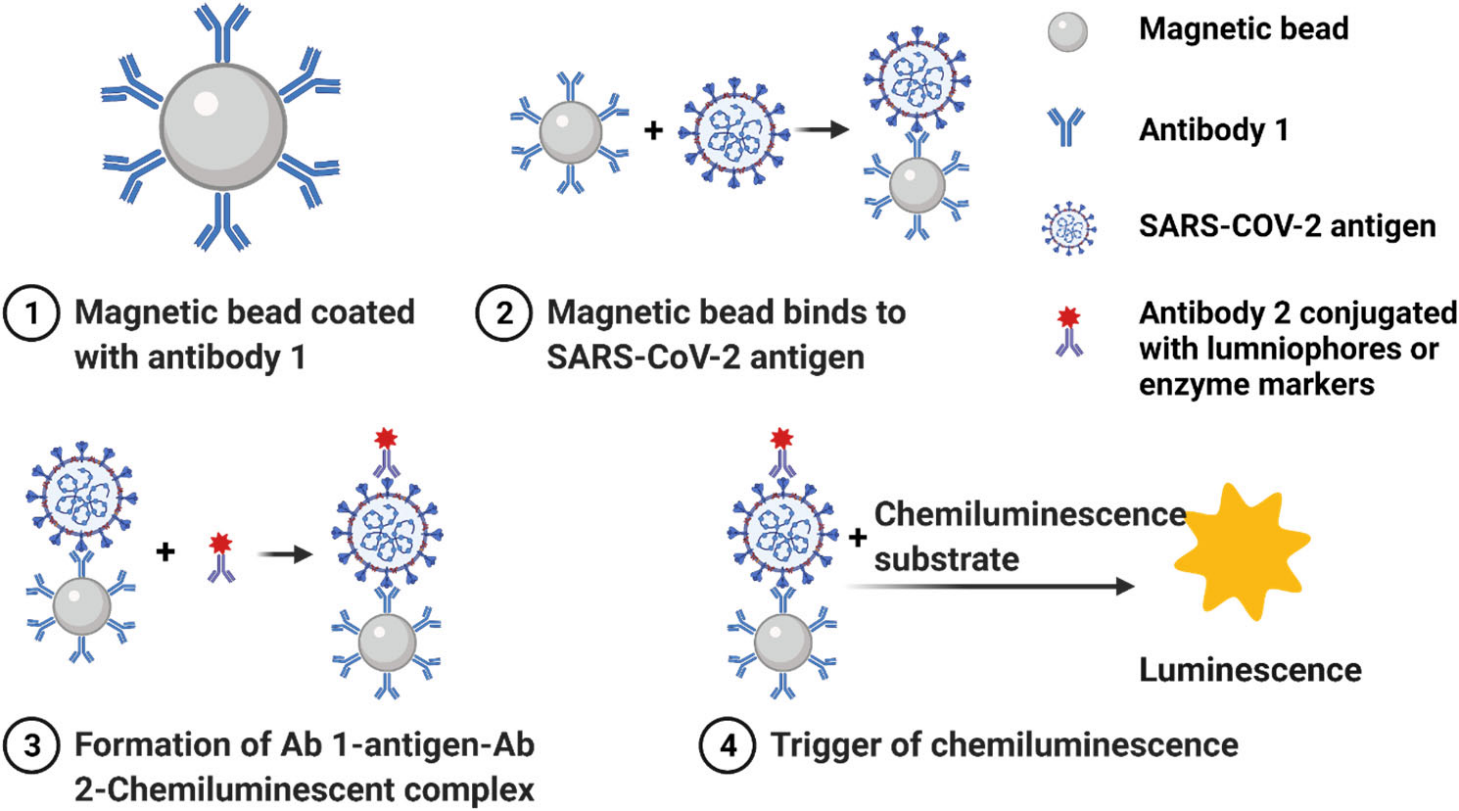
Mechanistic illustration of CLIA detecting SARS‐CoV‐2 antigen. This figure was created with BioRender.com.

Chemiluminesence makes use of a special type of chemical reaction, in which when the intermediates return from their excited state to their stable ground state, a photon is released and can be detected by the luminescent signal instrument.^[^ 58, 59 ^]^ Based on such a mechanism, chemiluminescence immunoassays (CLIA) is developed for quantitative antigen detection.^[^ ^59^ ^]^ In a typical CLIA, magnetic beads coated with anti‐COVID‐19 antibodies (Ab1) (**Figure** **7**a) can specifically bind to SARS‐CoV‐2 viral antigens in clinical samples (Figure 7b). The viral antigen then binds to another antibody (Ab2) conjugated with either lumninophore markers (such as acridinium and ruthenium esters) or enzyme markers (such as alkaline phosphatase and horseradish peroxidase with luminol)^[^ ^60^ ^]^ and later forms an Ab 1‐antigen‐Ab 2 immune complex **(**Figure 7c **).**The light emission is often times initiated by adding the pretrigger and/or trigger solutions **(**Figure 7d **).** SARS‐CoV‐2 antigens could be calibrated by the intensity of luminescence.^[^ ^61^ ^]^ The compatibility of multiplex tests for various biomarkers and the potential for high‐throughput automation are two of CLIA's primary advantages over traditional LFA.^[^ 62, 63 ^]^ CLIA may also be adaptable to a variety of test formats and is fast in data collecting with minimum noise interference.^[^ ^30^ ^]^

## 7 Electrochemical Assay

**Figure 8:**
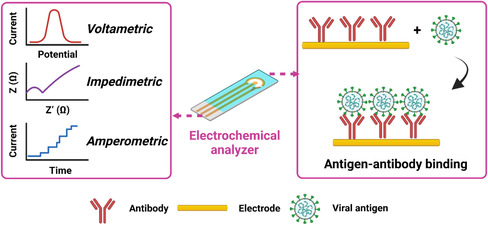
Principles of electrochemical techniques used for the detection of SARS‐CoV‐2 viral antigens. Inputs of antigen–antibody binding can be output in forms of voltametric, impedimetric, and amperometric signals via an electrochemical analyzer. This figure was created with BioRender.com.

In recent years, electrochemical sensors have been rapidly developed for detection of biological markers. Electrochemical signals can be classified as voltametric, impedimetric, and amperometric (**Figure** **8**). The electrical signals generated by the redox reactions between their recognition groups and target molecules on the electrode surface^[^ 40, 64 ^]^ (Figure 8) endow electrochemical sensors high specificity and sensitivity, advantages of both chemical reaction and electrochemical conduction.^[^ ^40^ ^.^ ^49^ ^]^ Unlike LFA and ELISA employing multiple antibodies, the electrochemical sensor‐based platform requires no complex labeling reagent.^[^ 34, 65 ^]^ This technology has been used in detection of COVID‐19 antigen with great performance manifested by low LoD and high specificity.^[^ ^35^ ^]^ Electrochemical sensors that are capable of distinguishing various forms of SARS‐CoV‐2 spike proteins have been reported.^[^ ^66^ ^]^ Compared with traditional ELISA and NA tests, electrochemical sensors have much faster readouts (within a few minutes) and lower manufacturing costs,^[^ 37, 67 ^]^ Furthermore, electrochemical assays can be easily integrated with digital analysis and big data collection using cloud‐connected mobile apps.^[^ 68, 69, 70 ^]^ Other advantages of the electrochemical assay include portability, less reagent consumption, and less preprocessing, with all well suited for POCT.^[^ 71, 72 ^]^ Taken together, the electrochemical sensor‐based platform is promising not only for COVID diagnosis but also for the detection of many other disease biomarkers.^[^ ^67^ ^]^

## 8 Surface Plasmon Resonance (SPR) Assay

**Figure 9:**
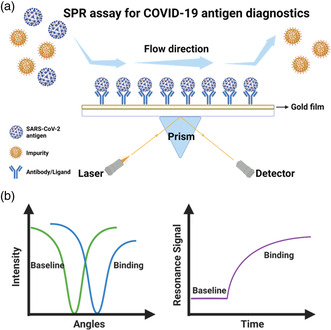
SPR setup for detection of COVID‐19 antigen. a) Schematic of SPR workflows. The antigens of interest are captured by antibodies bound to the gold substrate and can be detected by the change of laser signals. b) Sensorgram of an SPR measurement. Binding of antigen and antibody triggers shifts of laser intensity and resonance signal. This figure was created with BioRender.com.

The SPR assay enables rapid measurements of kinetics and affinity of bimolecular binding in real‐time and quantitative fashion.^[^ ^73^ ^]^ It is based on the electromagnetic resonance of the collective oscillations of free electrons occurring at a plasmonic metallic interface(i.e., a thin gold film)^[^ ^74^ ^]^ (**Figure** **9**a). This resonance intensity signal varies with the extent of the absorption of target molecules to ligands grafted onto the interface, which can be accurately detected and then converted to the quantification of molecules of interest^[^ ^75^ ^]^ (Figure 9b). The SPR test can transform tiny amounts of viral antigen into sensitive electrical signals, allowing for in situ and dynamic detection of virus presence and concentration.^[^ ^76^ ^]^ The SPR assay, in contrast to sophisticated and expensive NA assays, is a relatively simple and low‐cost procedure.^[^ ^77^ ^]^ In comparison with LFAs, the SPR assay provides superior LoD and sensitivity,^[^ ^78^ ^]^ which can be as low as 100 viral particles mL^−1^.^[^ ^79^ ^]^ This sensitivity is sufficient to classify most clinical samples, given that the median viral load for SARS‐CoV‐2 in nasal specimens was around 6 × 10^6^ copies mL^−1^.^[^ ^80^ ^]^ Briefly, as an antigen detection technology, the SPR assay has good simplicity and sensitivity and can be used as an effective alternative screening method to NA tests in evaluating large numbers of samples.^[^ ^81^ ^]^ However, its relatively high equipment dependence and reliance on fine processing of agents are currently prohibitive to POCT scenarios and in‐home use.^[^ ^82^ ^]^

## 9 Commercial COVID Antigen Test Kits in Western Countries

The outbreak of COVID‐19 has ushered in a period of rapid market expansion in the diagnostics industry. The global market for COVID‐19 diagnostic services was valued at $60.3 billion in 2020, with an estimated compound annual growth rate (CAGR) of > 15% during 2021–2027.^[^ ^83^ ^]^ Compared with the gold standard NA tests, antigen tests have much simpler operation and faster readout, with more flexible facility and operator requirements. Therefore, the development of novel commercial COVID‐19 antigen detection technology to supplement molecular testing is of great significance.

Here we summarized the commercial antigen test kits in the American and European markets. From October 2020 to April 2022, 48 antigen diagnostic tests for SARS‐CoV‐2, from 28 manufacturers, were granted Emergency Use Authorization (EUAs) by Food and Drug Administration (FDA) (**Table** **2**). Among them, 32 test kits are classical LFAs providing visual readouts, accounting for two‐thirds of all tests with EUAs. The remaining tests that require additional instruments to aid in the reading include ten fluorescence LFA test kits, four CLIA test kits, one electrochemical assay, and one SPR assay. The simplicity and rapidity of LFAs may be important reasons as to why they are favored by in vitro diagnostics (IVD) companies. In addition, 47 test kits target N protein, and there are only two products targeting RBD domains of S protein, including 1 N/S hybrid test kit. This is most likely due to the fact that S protein is less abundant and more prone to mutations under selective pressure than N protein.^[^ ^84^ ^]^ More importantly, many commercial test kits have satisfactory sensitivities (>90%) and specificities (>95%), with a considerable number of kits even claiming 100% specificity, and most test kits can provide results within 30 min.

**Table 2:** Commercial COVID‐19 antigen tests issued with U.S. FDA EUAs as of April 6, 2022

Product namea)	Entity	Date EUA issued or last updated	Mechanism	Target	Readout	PPA+NPA	Authorized settings	Effective time
Pilot COVID‐19 At‐Home Test	SD Biosensor, Inc.	04/04/2022	LFA	N protein	Visual	95.3%/100%	Home, H, M, W	20–30 min
iHealth COVID‐19 Antigen Rapid Test	iHealth Labs, Inc.	04/04/2022	LFA	N protein	Visual	94.3%/98.1%	Home, H, M, W	15–30 min
BinaxNOW COVID‐19 Antigen Self Test	Abbott Diagnostics Scarborough, Inc.	04/04/2022	LFA	N protein	Visual	84.6%/98.5%	Home, H, M, W	15–30 min
MaximBioClearDetect COVID‐19 Antigen Home Test	Maxim Biomedical, Inc.	03/30/2022	LFA	N protein	Visual	86.9%/98.9%	Home, H, M, W	15–30 min
iHealth COVID–19 Antigen Rapid Test	iHealth Labs, Inc.	03/29/2022	LFA	N protein	Visual	94.3%/98.1%	Home, H, M, W	15–30 min
BD Veritor At‐Home COVID–19 Test	Becton, Dickinson and Company	03/25/2022	LFA	N protein	Digital	84.6%/99.8%	Home, H, M, W	>20 min
CareStart COVID‐19 Antigen Home Test	Access Bio, Inc.	03/25/2022	LFA	N protein	Visual	87.0%/98.0%	Home, H, M, W	10–15 min
CelltrionDiaTrust COVID‐19 Ag Home Test	Celltrion USA, Inc.	03/23/2022	LFA	N & S protein	Visual	86.7%/99.8%	Home, H, M, W	15–20 min
CLINITEST Rapid COVID‐19 Antigen Self–Test	Siemens Healthineers	03/23/2022	LFA	N protein	Visual	86.5%/99.3%	Home, H, M, W	15‐20 min
Ellume COVID–19 Home Test	Ellume Limited	03/18/2022	LFA (fluorescence)	N protein	Digital	96.0%/100%	Home, H, M, W	15 min
INDICAID COVID‐19 Rapid Antigen At‐Home Test	PHASE Scientific International, Ltd.	03/16/2022	LFA	N protein	Visual	81.7%/99.4%	Home, H, M, W	20–25 min
Atellica IM SARS‐CoV‐2 Antigen (CoV2Ag)	Siemens Healthcare Diagnostics, Inc.	03/11/2022	CLIA	N protein	Digital	85.1%/100%	H, M	>24 min
ADVIA Centaur SARS‐CoV‐2 Antigen (CoV2Ag)	Siemens Healthcare Diagnostics, Inc.	03/11/2022	CLIA	N protein	Digital	85.1%/100%	H, M	>24 min
Clip COVID Rapid Antigen Test	Luminostics, Inc.	03/04/2022	LFA (fluorescence)	N protein	Digital	96.9%/100%	H, M, W	>30 min
SCoV‐2 Ag Detect Rapid Test	InBios International, Inc.	03/03/2022	LFA	N protein	Visual	86.7%/100%	Home, H, M, W	20–25 min
SCoV‐2 Ag Detect Rapid Self‐Test	InBios International, Inc.	03/03/2022	LFA	N protein	Visual	85.7%/100%	H, M, W	20–25 min
ASSURE‐100 Rapid COVID‐19 Test	Oceanit Foundry LLC	02/28/2022	LFA	N protein	Visual	89.0%/100%	H, M, W	20–30 min
INDICAID COVID‐19 Rapid Antigen Test	PHASE Scientific International, Ltd.	02/22/2022	LFA	N protein	Visual	86.7%/97.2%	H, M, W	20–25 min
Flowflex COVID‐19 Antigen Home Test	ACON Laboratories, Inc	02/18/2022	LFA	N protein	Visual	92.0%/100%	Home, H, M, W	15–30 min
LumiraDx SARS‐CoV‐2 Ag Test	LumiraDx UK Ltd.	02/17/2022	LFA	N protein	Digital	97.6%/96.6%	H, M, W	~12 min
LIAISON SARS‐CoV‐2 Ag	DiaSorin, Inc.	02/16/2022	CLIA	N protein	Digital	84.4%/99.5%	H, M	120‐180 min
BinaxNOW COVID‐19 Ag Card	Abbott Diagnostics Scarborough, Inc.	02/04/2022	LFA	N protein	Visual	84.6%/98.5%	Home, H, M, W	15–30 min
BinaxNOW COVID‐19 Ag Card Home Test	Abbott Diagnostics Scarborough, Inc.	02/04/2022	LFA	N protein	Visual	84.0%/98.3%	H, M, W	15–30 min
Nano‐Check COVID‐19 Antigen Test	Nano‐Ditech Corp.	02/01/2022	LFA	N protein	Visual	90.3%/100%	H, M, W	15–20 min
InteliSwab COVID‐19 Rapid Test Rx	OraSure Technologies, Inc.	01/27/2022	LFA	N protein	Visual	85.0%/98.0%	Home, H, M, W	30–40 min
InteliSwab COVID‐19 Rapid Test	OraSure Technologies, Inc.	01/27/2022	LFA	N protein	Visual	85.0%/98.0%	Home, H, M, W	30–40 min
InteliSwab COVID‐19 Rapid Test Pro	OraSure Technologies, Inc.	01/27/2022	LFA	N protein	Visual	85.0%/98.0%	Home, H, M, W	30–40 min
iHealth COVID‐19 Antigen Rapid Test Pro	iHealth Labs, Inc.	01/14/2022	LFA	N protein	Visual	88.2%/100%	H, M, W	15–30 min
Simoa SARS‐CoV‐2 N Protein Antigen Test	Quanterix Corporation	12/21/2021	LFA	N protein	Visual	83.9%/99.9%	H, M	10–20 min
Sienna‐Clarity COVID‐19 Antigen Rapid Test Cassette	Salofa Oy	12/17/2021	LFA	N protein	Visual	87.5%/98.9%	H, M, W	10–20 min
BD Veritor System for Rapid Detection of SARS‐CoV‐2	Becton, Dickinson and Company	12/10/2021	LFA (fluorescence)	N protein	Digital	84.0% /100%	H, M, W	15–20 min
CareStart COVID‐19 Antigen Test	Access Bio, Inc.	12/02/2021	LFA	N protein	Visual	93.4%/99.3%	H, M, W	10–15 min
GenBody COVID‐19 Ag	GenBody Inc.	11/17/2021	LFA	N protein	Visual	91.1%/100%	H, M, W	15–20 min
VITROS Immunodiagnostic Products SARS‐CoV‐2 Antigen Reagent Pack	Ortho Clinical Diagnostics, Inc.	11/16/2021	CLIA	N protein	Digital	80.0%/100%	H, M	>48 min
QuickVue SARS Antigen Test	Quidel Corporation	11/09/2021	LFA	N protein	Visual	96.8%/99.1%	H, M, W	10–15 min
Status COVID‐19/Flu A&B	Princeton BioMeditech Corp.	10/27/2021	LFA	N protein	Visual	93.1%/100%	H, M, W	15–20 min
QuickVue At‐Home OTC COVID‐19 Test	Quidel Corporation	10/21/2021	LFA	N protein	Visual	83.5%/99.2%	Home, H, M, W	10–15 min
SPERA COVID‐19 Ag Test	Xtrava Health	10/12/2021	LFA	N protein	Visual	91.8%/96.9%	H, M, W	15‐30 min
NIDS COVID‐19 Antigen Rapid Test Kit	ANP Technologies, Inc	09/24/2021	LFA	N protein	Visual	95.1%/97.0%	H, M, W	15–30 min
CelltrionDiaTrust COVID‐19 Ag Rapid Test	Celltrion USA, Inc.	09/01/2021	LFA	N protein	Visual	93.3%/99.0%	H, M, W	15–20 min
QIAreach SARS‐CoV‐2 Antigen	QIAGEN GmbH	08/05/2021	LFA (fluorescence)	N protein	Digital	85.0%/99.1%	H, M	2–15 min
ellume.lab COVID Antigen Test	Ellume Limited	07/08/2021	LFA (fluorescence)	N protein	Digital	81.8%/100%	H, M, W	3–15 min
Sofia SARS Antigen FIA	Quidel Corporation	06/11/2021	LFA (fluorescence)	N protein	Digital	96.7%/100%	H, M, W	~15 min
Omnia SARS‐CoV‐2 Antigen Test	Qorvo Biotechnologies, LLC.	04/13/2021	SPR assay	N protein	Digital	89.5%/100%	H, M	15–20 min
BD Veritor System for Rapid Detection of SARS‐CoV‐2 & Flu A+B	Becton, Dickinson and Company	03/24/2021	LFA (fluorescence)	N protein	Digital	86.7%/99.5%	H, M, W	15–20 min
QuickVue At‐Home COVID‐19 Test	Quidel Corporation	03/01/2021	LFA	N protein	Visual	84.8%/99.1%	Home, H, M, W	10–15 min
Sampinute COVID‐19 Antigen MIA	Celltrion USA, Inc.	10/23/2020	Electrochemical assay	S protein	Digital	94.4%/100%	H, M	>40 min
Sofia 2 Flu + SARS Antigen FIA	Quidel Corporation	10/02/2020	LFA (fluorescence)	N protein	Digital	95.2%/100%	H, M, W	~15 min

a) Products are listed in a sequence of the date EUA issued or last updated. This table is updated from our published work^[^ ^90^ ^]^ and the U.S. FDA website.^[^ ^122^ ^]^ Effective time is counted from the contact of swab sample and buffer. PPA and NPA stand for positive percentage agreement and negative percentage agreement with NA test thus representing sensitivity and specificity of a test, respectively. N protein: nucleocapsid protein. S protein: Spike protein. H: Laboratories certified under the Clinical Laboratory Improvement Amendments of 1988 (CLIA) that meet requirements to perform high complexity tests. M: Laboratories certified under the Clinical Laboratory Improvement Amendments of 1988 (CLIA) that meet requirements to perform moderate complexity tests. W: Patient care settings operating under a CLIA Certificate of Waiver.

**Figure 10:**
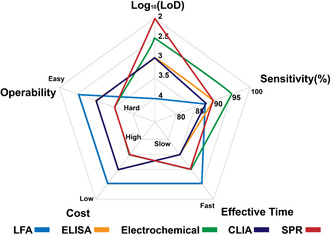
Comparison of current antigen test kits with EUAs in the U.S. using a five‐star chart model. LFA, ELISA, CLIA, and SPR assay are expressed in blue, orange, green, purple, and pink colors, respectively. The unit of LoD is viral copies mL^−1^. Note: These five‐star dimensions are qualitative or semiquantitative statistics of each test principle obtained from the U.S. FDA website^[^ ^122^ ^]^ and might vary with specific commercial products.

Features including LoD, sensitivity, effective time, cost, and operability of test kits in the U.S. market are summarized using a five‐star chart model (**Figure** **10**). Generally, LFA is obviously superior to all other test methods in terms of effective time, cost, and operability, with compromised LoD and sensitivity. Among five methods, ELISA and CLIA exhibit intermediate levels of LoD, sensitivity, cost, and operability. Despite relatively longer assay time required, satisfactory parameters make these two methods as standard protocols in R&D labs but much less popular in self‐testing and POC scenarios. Electrochemical and SPR assays present excellent LoD and sensitivities but their commercial competitiveness is hampered by their high cost, long reaction time, and complex operations. One should recognize that the LoD and sensitivities of the commercial test kits were not necessarily correlated. A possible explanation is that the LoDs were measured with inactivated viruses while the sensitivities were measured with clinic specimens. Particularly, various manufacturers of inactivated viruses might have different quality control standards, which could subsequently lead to different levels of specific antigens arising from unassembled viral particles and different tissue culture infectious dose (TCID) assay results.^[^ ^85^ ^]^ Proteins and polysaccharidein saliva and mucus from different individuals could affect the afterward clinic performance as well.^[^ ^86^ ^]^

**Figure 11:**
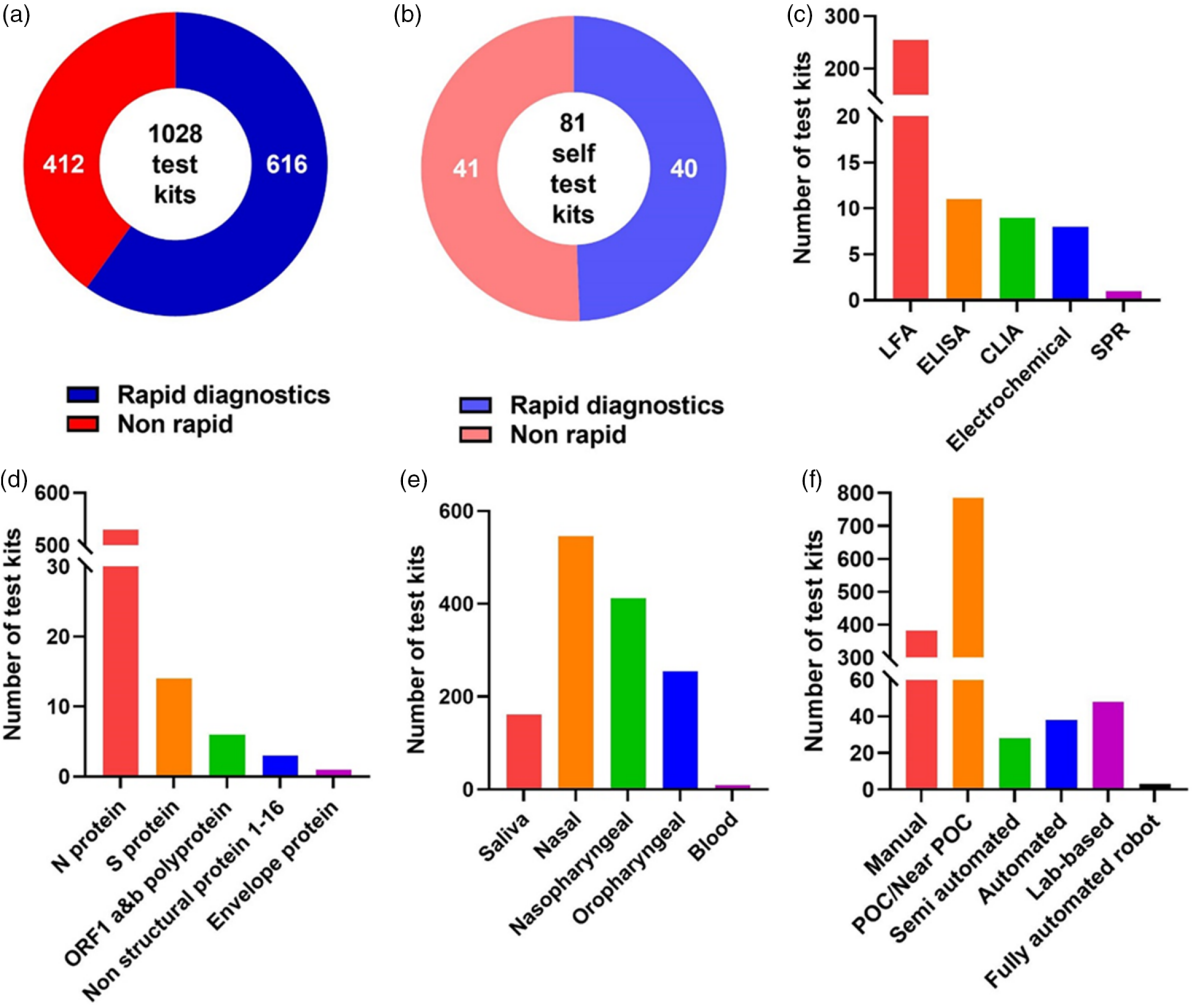
Statistics of COVID‐19 antigen tests issued with CE marking in European Economic Area as of April 6, 2022. All data were collected from the COVID‐19 In Vitro Diagnostic Devices and Test Methods Database.^[^ ^123^ ^]^ a) Total number of antigen test kits. b) Total number of antigen test kits classified as self‐test. c) Number of various kits categorized by the European Commission based on detection principle. d) Number of various kits categorized by the European Commission based on target. e) Number of various kits categorized by the European Commission based on specimen. f) Number of various kits categorized by the European Commission based on test format. Note: Statistical classifications in (d–f) are not exclusive as some test kits might apply to multiple targets, specimen, or test formats.

During the same period, 1028 antigen test kits were approved with CE marking by the European Commission, including 616 rapid test kits and 412 nonrapid ones (**Figure** **11**a). Among them, 40 rapid test kits and 41 nonrapid test kits were authorized for self‐testing use (Figure 11b). The big difference in numbers of approved antigen test kits may be attributed to the differences of medical regulations, insurances, and government guidelines between the two largest pharmaceutical markets in the world. Indeed, the COVID‐19 diagnosis in the U.S. depended more on NA tests, while the European governments (Britain, Germany, etc.) emphasized the importance of antigen detection much earlier. Among products officially revealing their test principles, 255 kits were based on LFA, accounting for the vast majority of the pool. Numbers of ELISA, CLIA, Electrochemical, and SPR assays were 11, 9, 8, and 1, respectively (Figure 11c). The European market share of LFA products was similar to that in U.S. as the convenience of LFA was well acknowledged by both markets. Interestingly, the antigen targets and test specimens in the European market are more diverse (Figure 11d,e). Benefiting from the supportive regulatory policies of the European Commission, most of these products first appeared in the early stage of the pandemic, revealing that antigen tests could quickly and flexibly respond to the epidemic.

According to FDA statistics, one major concern for antigen testing kits that rely on visual observations is that their sensitivities are generally lower than those that rely on machine reading despite some exceptions perhaps employing higher affinity antibodies and/or fine labeling arts. One solution is to provide better interpretation tools or essential auxiliary devices to convert visual signals to digital readout, which may improve assay LoD and sensitivity. Many European test kits had already been engaged in such attempts that nearly 70 products utilized semiautomated, automated, or even robot‐assisted assays (Figure 11f). Devises or technologies that involve smart phones also facilitate in‐home data collection and result interpretation. An alternative approach is to use bispecific monoclonal antibodies to boost up the signal and improve sensitivity.^[^ ^87^ ^]^ Concerns have been raised whether rapid antigen tests for SARS‐CoV‐2 can result in false‐positive test results and undermine pandemic management for COVID‐19. Emerging evidence suggested that the overall rate of false‐positive results among the total rapid antigen test screens for SARS‐CoV‐2 was very low.^[^ 88, 89 ^]^ However, false‐positive results can occur, especially when used in situations where the prevalence of infection is low, which is true for all in vitro diagnostic tests.

The sample loading volumes in most commercial tests are usually low (i.e., several drops),^[^ ^90^ ^]^ because their capillary or microchannel structures relying on passive diffusion to process samples can be easily saturated by excessive fluid. The intrinsic defect significantly slows detection and increases the possibility of false‐negative results. Combining ultrahigh‐throughput hydrodynamic filtration and sandwich immunoassay is one promising solution for increasing sample loading volume.^[^ ^90^ ^]^ Indeed, in our recent study, using a simple handheld injection, the microfluidic test kit was able to process fluid samples on a milliliter scale, a volume that was 1–2 orders of magnitude greater than that of conventional methods resulting in improved sensitivity.^[^ ^90^ ^]^

The emergence of SARS‐CoV‐2 variants has also raised concerns about evasion of detection by rapid antigen diagnostics. Indeed, mutation of N protein in SARS‐CoV‐2 VoCs such as D399N and T135I may potentially lead to false‐negative results in rapid antigen tests, despite a high viral load.^[^ 91, 92 ^]^ Polyclonal anti‐N antibodies have been shown sensitive against various mutants including N501Y, H69/V70, D796H, and D614G.^[^ 93, 94 ^]^ When a single monoclonal antibody is used for capturing or labeling, it is of greater concern whether the performance of the test could be altered by emerging strains of SARS‐CoV‐2. On the other hand, the FDA requests that antigen test manufacturers report surveillance when an existing test becomes invalid due to a new viral variant.

To expedite identification and isolation of infected cases, COVID‐19 antigen self‐testing has been implemented worldwide. The advantages for antigen testing over NA testing are obvious. Most antigen tests are easy to distribute and could be carried out in home, while NA testing relies on a PCR laboratory, trained professionals, and sophisticated testing procedures, which are further complicated by pooled assays in some highly populated epicenters such as Shanghai.^[^ ^95^ ^]^ The individuals in such epicenters have to wait in queues for NA tests, taking the risk of being infected on site, while in‐home antigen self‐testing minimizes the chance of viral exposure. According to studies, more frequent screening reduces the likelihood of an outbreak, and the fast diagnostic turnaround time of an antigen test tends to outweigh the reduced sensitivity of NA testing.^[^ ^96^ ^]^ Because antigen testing is simple, low cost, and immediate, massive rapid‐testing programs have been implemented in public places such as schools, hospitals, prisons, airports, borders, workplaces, tourist attractions, and parks. In those crowded places, we envision flexible transoral robot adopted for COVID‐19 swab sampling, reducing the risk of infection while also ensuring accurate swab sampling.^[^ 88, 89 ^]^ The quarantine and isolation policy for individuals who are antigen‐tested positive varies by countries. Authorities in some countries require an immediate NA test to confirm infection. Currently, certified laboratories and testing sites are committed to report all positive cases to the state or local public health departments. An effective report system for positive cases in antigen self‐test is important for public health surveillance and to avoid community transmission of COVID‐19.

## 10 Conclusion and Outlook

Antigen detection provides a number of distinct advantages: 1) high speed, low cost, and noninvasive small volumes of sampling;^[^ ^97^ ^]^ 2) equipment‐free interpretation of qualitative results and mild equipment dependence to interpret semiquantitative results;^[^ ^98^ ^]^ 3) relatively short R&D cycles and quick market‐oriented iterations;^[^ ^99^ ^]^ 4) easy sampling and simple testing procedures;^[^ ^100^ ^]^ 5) universal test objects and scenarios;^[^ ^101^ ^]^ and 6) long shelf‐life and easily achievable storage conditions.^[^ ^102^ ^]^

Antigen tests remain to be further improved in many aspects: 1) inaccurate sample volume affects accuracy (especially in LFA products with manual sample loading);^[^ ^103^ ^]^ 2) small sample volume can limit LoD and sensitivity;^[^ ^90^ ^]^ 3) antigen–antibody binding signal barely undergoes a secondary amplification like in NA tests; thus, trace amounts of antigen could become undetectable;^[^ ^104^ ^]^ 4) time to results is usually uncertain and varies by the volume, viscosity, concentration, etc. of the sample (Table 1); and 5) the fact that antigen–antibody testing relies on macroscopic effects (aggregation, color change, current change, etc.) is problematic due to the intrinsic scarcity of antigen proteins of SARS‐CoV‐2 viruses.^[^ ^105^ ^]^ Therefore, the test sensitivity can be uncertain as the virus load gradually drops to a low concentration, which usually occurs in the first 5–7 days before symptom onset and the late postsymptom period.^[^ ^106^ ^]^ Nevertheless, daily screening could significantly increase the chance of detection.^[^ ^107^ ^]^

We envision the following future technical developments of current COVID‐19 antigen diagnostic platforms: 1) acceleration of antigen design and high‐throughput screening;^[^ ^108^ ^]^ 2) innovation of multiplexed platforms for simultaneous detection of SARS‐CoV‐2 and other pathogens;^[^ ^109^ ^]^ 3) combinations of technologies like lab‐on‐a‐chip,^[^ ^110^ ^]^ machine learning,^[^ ^111^ ^]^ and cloud methods^[^ ^112^ ^]^ to increase data collection and reduce misinterpretation of results; 4) improvement of quality controls and manufacturing practices to meet the increasing demand for testing;^[^ ^113^ ^]^ and 5) customization of usable and affordable test kits for individuals with disabilities^[^ 114, 115 ^]^ (i.e., color blindness, impaired mobility) and areas with inadequate medical infrastructure.^[^ ^90^ ^]^

With its accuracy, cost effectiveness, speed, and simplicity, we look forward to antigen testing continuing to play an important role in the battle against the COVID‐19 pandemic.

## Conflict of Interest

The authors declare no conflict of interest.
